# Intercellular Signaling Pathway among Endothelia, Astrocytes and Neurons in Excitatory Neuronal Damage

**DOI:** 10.3390/ijms14048345

**Published:** 2013-04-16

**Authors:** Takako Takemiya, Kanato Yamagata

**Affiliations:** 1 Medical Research Institute, Tokyo Women’s Medical University, Shinjuku, Tokyo 162-8666, Japan; 2 Neural Plasticity Project, Tokyo Metropolitan Institute of Medical Science, Tokyo 156-8506, Japan; E-Mail: yamagata-kn@igakuken.or.jp

**Keywords:** microsomal prostaglandin E synthase-1 (mPGES-1), prostaglandin E_2_ (PGE_2_), endothelial cell, EP3, kainic acid, Ca^2+^ levels, astrocyte, neuronal damage

## Abstract

Neurons interact closely with astrocytes via glutamate; this neuron-glia circuit may play a pivotal role in synaptic transmission. On the other hand, astrocytes contact vascular endothelial cells with their end-feet. It is becoming obvious that non-neuronal cells play a critical role in regulating the neuronal activity in the brain. We find that kainic acid (KA) administration induces the expression of microsomal prostaglandin E synthase-1 (mPGES-1) in venous endothelial cells and the prostaglandin E_2_ (PGE_2_) receptor prostaglandin E receptor (EP)-3 on astrocytes. Endothelial mPGES-1 exacerbates KA-induced neuronal damage in *in vivo* experiments. In *in vitro* experiments, mPGES-1 produces PGE_2_, which enhances astrocytic Ca^2+^ levels via the EP3 receptor and increases Ca^2+^-dependent glutamate release, thus aggravating neuronal injury. This novel endothelium-astrocyte-neuron signaling pathway may be crucial for driving neuronal damage after repetitive seizures and could be a new therapeutic target for epilepsy and other brain disorders.

## 1. Introduction

Prostaglandin E_2_ (PGE_2_) is one of the most important modulators in inflammation. In the brain, PGE_2_ is also involved in pathological processes, such as fever, seizure and cerebral ischemia [1–3], suggesting that these processes might be associated with inflammation. PGE_2_ is sequentially synthesized from arachidonic acid by cyclooxygenase (COX) and PGE_2_ synthase (PGES) in various cells and tissues. COX catalyzes the first step in the synthesis of prostaglandins (PGs) and exists in two homologous isoforms. One isoform is the constitutively active COX-1, which is widely distributed in various cell types and is thought to mediate physiological responses. The other isoform is the inducible COX-2, which is expressed in several cell types in response to various stimuli, such as neuronal activity, cytokines and pro-inflammatory molecules [4–8]. Inducible COX-2 expression in the brain is associated with acute neurotoxicity, such as seizures and ischemia [2,3,9,10]. COX-2 is also involved in delayed pro-inflammatory activities, which aggravate the neuronal damage found in neurodegenerative diseases, such as amyotrophic lateral sclerosis (ALS), Parkinson’s disease (PD), multiple sclerosis (MS) and Alzheimer’s disease (AD) [9,11]. We find that COX-2 is induced in non-neuronal cells late after seizure and facilitates neuronal loss in the hippocampus [12].

In this review, we first focus on the co-induction of COX-2 and microsomal prostaglandin E synthase-1 (mPGES-1), an enzyme downstream of COX-2, in brain endothelial cells after seizures. Next, we provide a view on the role of endothelial mPGES-1 in neuronal loss in the hippocampus. Finally, we will present a novel mechanism for exacerbation of neuronal damage by PGE_2_ derived from endothelial mPGES-1 and discuss the intercellular signaling pathway among endothelia, astrocytes and neurons in this process.

## 2. Role of Endothelial mPGES-1 in KA-Induced Neuronal Damage

### 2.1. Co-Induction of COX-2 and mPGES-1 in Brain Endothelial Cells

**Figure 1:**
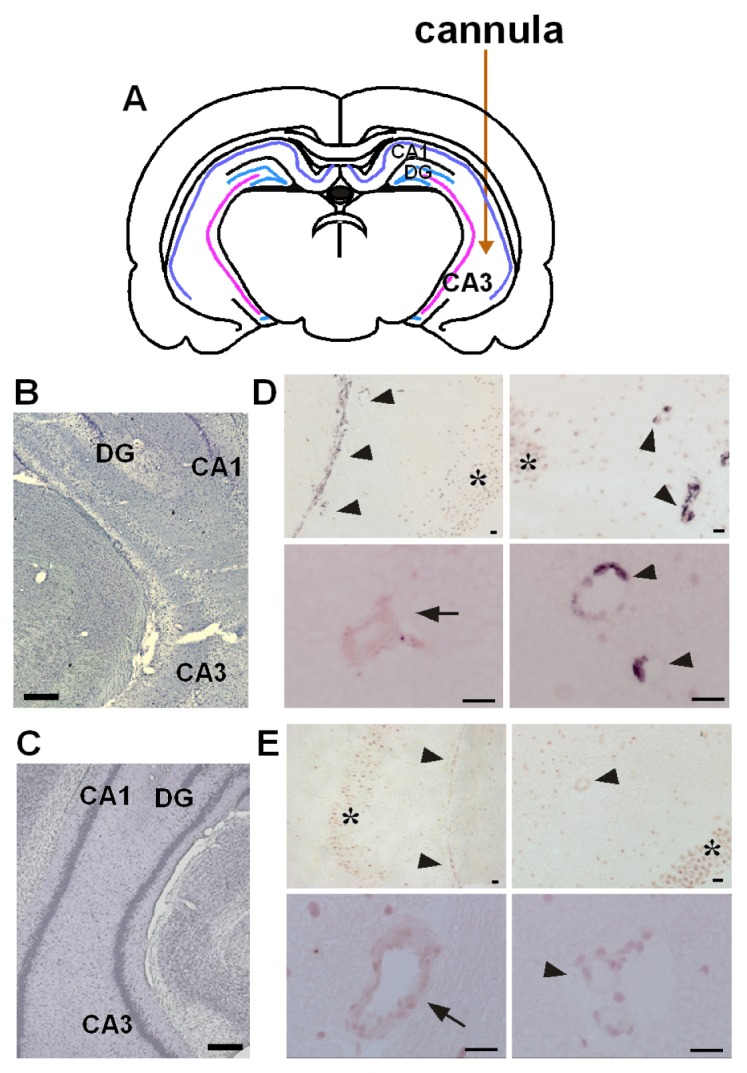
Neuronal injury elicited by kainic acid (KA) and microsomal prostaglandin E synthase-1 (mPGES-1) mRNA induction in the hippocampus following KA microinjection (summarized from ref. [12] and [19]). (**A**) Placement of cannula tips for microinjection into the hippocampal CA3 region. (**B** and **C**) KA-injected hippocampus showing marked cell loss in the ipsilateral side of the CA3 region (**B**), but little cell loss in the contralateral side (**C**). Scale bars: 400 μm. (**D** and **E**) mPGES-1 mRNA levels were increased in the veins (arrowheads), but not in the arteries (arrows), neurons (asterisks) or other cells on the ipsilateral side (**D**) or in any cells on the contralateral side (**E**) at 8 h after KA injection. Scale bars: 20 μm.

**Figure 2:**
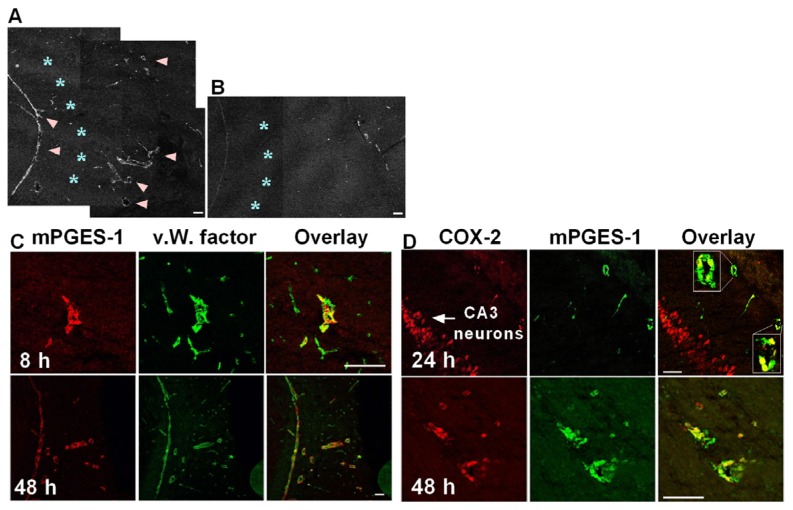
Induction of the mPGES-1 protein in the hippocampus following KA microinjection (summarized from ref. [19]). (**A** and **B**) Immunostaining of the mPGES-1 protein in the hippocampal CA3 region at 24 h after KA (**A**) or PBS (**B**) injection. mPGES-1 appeared in the veins (arrowheads), but not in the CA3 neurons (asterisks). (**C**) Double immunostaining of mPGES-1 and von Willebrand (v.W.) factor at 8 h and 48 h after KA treatment; (**D**) Double immunostaining for COX-2 and mPGES-1 at 24 h and 48 h after KA injection. Scale bars: 100μm.

We were the first to demonstrate that PGE_2_ is synthesized by mPGES-1 coupling with COX-2 in brain endothelial cells in lipopolysaccharide (LPS)-induced fever [13], and subsequent publications have confirmed that mPGES-1 is co-induced with COX-2 during fever or inflammation [14–17]. Furthermore, mPGES-1 is induced in the hippocampus after epileptic seizures caused by kainic acid (KA) microinjection. KA is an analogue of the excitatory amino acid glutamate and is suited for research to investigate the mechanisms for hippocampal neuronal loss after seizures, because KA induces generalized convulsion and causes neuronal damage in the hippocampus several days after seizures [18]. Unilateral KA microinjection causes neuronal loss in the injection side, but not in the contralateral side (Figure 1A–C) [12]. Kainate induces *mPGES-1* mRNA in the veins, but not in the arteries, neurons or other cells on the ipsilateral side (Figure 1D) or in any cells on the contralateral side (Figure 1E), at 8h after KA microinjection [19]. The mPGES-1 protein is localized in the structure of blood vessel, but not in other cells, such as neurons or glial cells, 24 h after KA injection (Figure 2A,B) [19]. Double-immunostaining for both mPGES-1 and von Willebrand (v.W.) factor (an endothelial cell marker) shows that mPGES-1 is induced in endothelial cells (Figure 2C). In addition, the co-induction of mPGES-1 and COX-2 in the endothelial cells was found to continue up to 48 h after the microinjection (Figure 2D) [19].

### 2.2. Role of Endothelial mPGES-1 in Hippocampal Neuronal Loss

**Figure 3:**
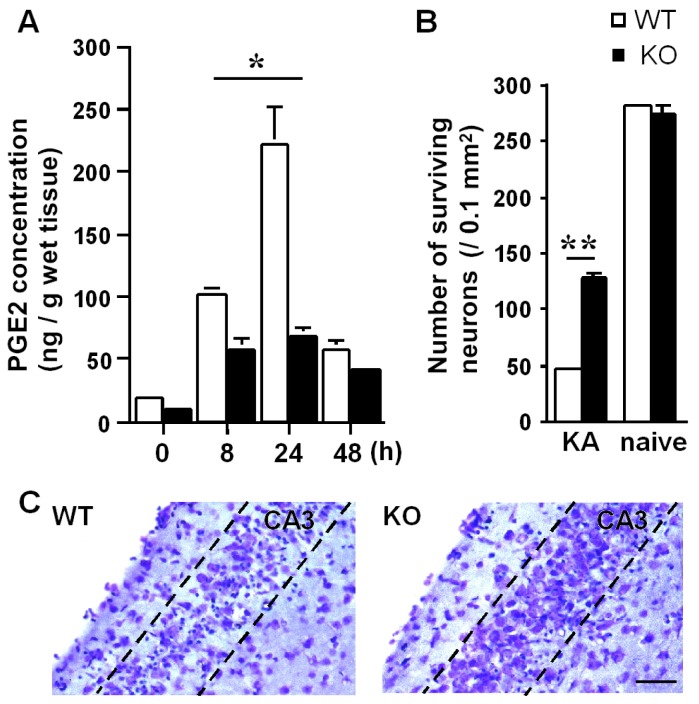
Inducible mPGES-1 produces prostaglandin E_2_ (PGE_2_) and stimulates neuronal damage (summarized from ref. [19]). (**A**) PGE_2_ concentration in the hippocampi of wild-type (WT) mice (*n* = 6–8) and *mPGES-1*^−/−^ mice (*n* = 6–8) following KA injection; (**B**) Under naive conditions, there was no significant difference in neuronal density between the WT (*n* = 7) and *mPGES-1*^−/−^ mice (*n* = 6), whereas a significant difference was observed in the number of neurons between the WT (*n* = 7) and *mPGES-1*^−/−^ mice (*n* = 7) at 48 h after KA microinjection; (**C**) Nissl staining of the hippocampal CA3 region of the WT (left) and *mPGES-1*^−/−^ mice (right) at 48 h after KA microinjection. *p < 0.01, **p < 0.005, Scale bars: 100 μm.

We also addressed whether mPGES-1 has an effect on neuronal death after KA-induced seizure by using *mPGES-1*^−/−^ mice [20]. Our results revealed that endothelial mPGES-1 is a key enzyme for the production of PGE_2_ (Figure 3A), which stimulates neuronal cell death (Figure 3B,C) [19]. Although it is widely thought that brain PGE_2_ is synthesized in and derived from astrocytes [21–23], microglia [24] and neurons [25], we found that most brain PGE_2_, which affects neuronal death, is supplied by endothelial mPGES-1. These findings raise the question of how endothelial PGE_2_ regulates neuronal damage. Next, we propose a mechanism for the stimulating role of endothelial PGE_2_ in neuronal death and then verify our hypothesis step by step.

## 3. Mechanism for Exacerbation of Neuronal Damage by Endothelial mPGES-1

### 3.1. Hypothetical Mechanism for Exacerbation by mPGES-1

Brain endothelial cells are surrounded by astrocytic end-feet [26], suggesting that the PGE_2_ produced in endothelial cells may have a direct effect on the adjacent astrocytes. Several lines of evidence indicate that prostaglandin E receptor (EP) receptors are present on cultured astrocytes. In addition, exogenous PGE_2_ immediately evokes Ca^2+^-dependent glutamate release from astrocytes [27]; therefore, astrocytes may be activated by endogenous PGE_2_ to elevate the intracellular Ca^2+^ ([Ca^2+^]_i_) levels directly through the PGE_2_ receptor. Moreover, astrocytes can modulate synaptic transmission through the release of glutamate [28–30], which may play a crucial role in delayed neuronal injury after seizures [31]. Taken together, we hypothesize that the endothelial PGE_2_ produced by mPGES-1 directly activates EP receptors on astrocytes, elevating the astrocytic [Ca^2+^]_i_ levels and, subsequently, evoking sustained glutamate release, ultimately leading to neuronal damage. To investigate the mechanisms of the neuronal damage while maintaining the intercellular associations among endothelial cells, astrocytes and neurons, we used hippocampal slice culture prepared from wild-type (WT) and *mPGES-1*^−/−^ mice.

### 3.2. Increases in Hippocampal PGE_2_ Concentration and Astrocytic Ca^2+^ Levels after KA Treatment

**Figure 4:**
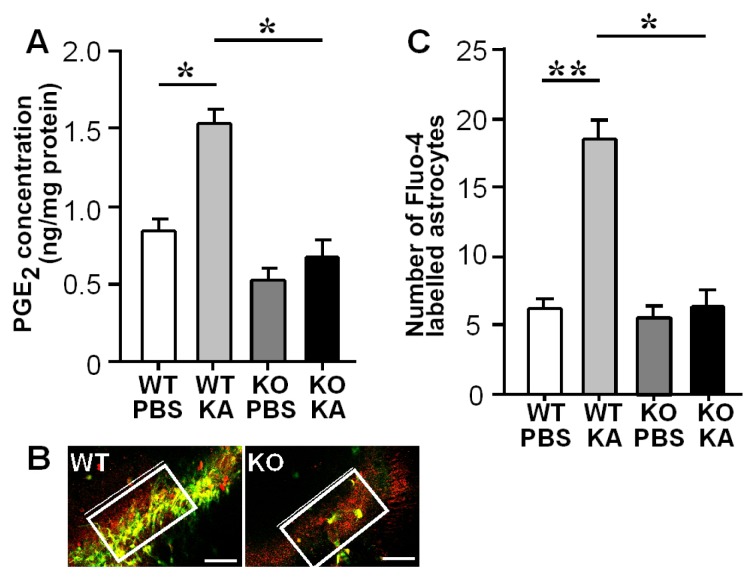
KA-induced mPGES-1 produces PGE_2_ and increases the astrocytic Ca^2+^ levels, promoting glutamate release from astrocytes and causing neuronal damage (summarized from ref. [32]). (**A**) PGE_2_ concentrations in cultured slices from WT and *mPGES-1*^−/−^ mice after the addition of KA or PBS for 17 h (*n* = 5–6); (**B**) Ca^2+^ imaging in the WT (left) and *mPGES-1*^−/−^ slices (right); (**C**) The number of Fluo-4 labeled astrocytes within the rectangular areas in WT and *mPGES-1*^−/−^ slices treated with KA or PBS (*n* = 5). *p < 0.001, **p < 0.0001.

First, we treated the WT hippocampal slices with KA, which significantly elevated the PGE_2_ concentration in the slices. This increase in PGE_2_ was not observed in the *mPGES-1*^−/−^ slices (Figure 4A). Next, we labeled [Ca^2+^]_i_ and astrocytes in the slices with Fluo-4 (Ca^2+^ indicator) and sulforhodamine101 (SR101; astrocyte marker), respectively (Figure 4B), and counted the number of astrocytes with elevated [Ca^2+^]_i_ levels within a set rectangular area of the hippocampal CA3 region (Figure 4C). The number of these cells within the rectangular area was significantly smaller in the *mPGES-1*^−/−^ slices (Figure 4D, right panel) than in the WT slices (Figure 4D, left panel and Figure 4E). These results suggest that the PGE_2_ derived from mPGES-1 upregulates the astrocytic [Ca^2+^]_i_ levels in the hippocampus, especially in the CA3 region.

### 3.3. Activation of Astrocytic EP3 Receptors by KA

**Figure 5:**
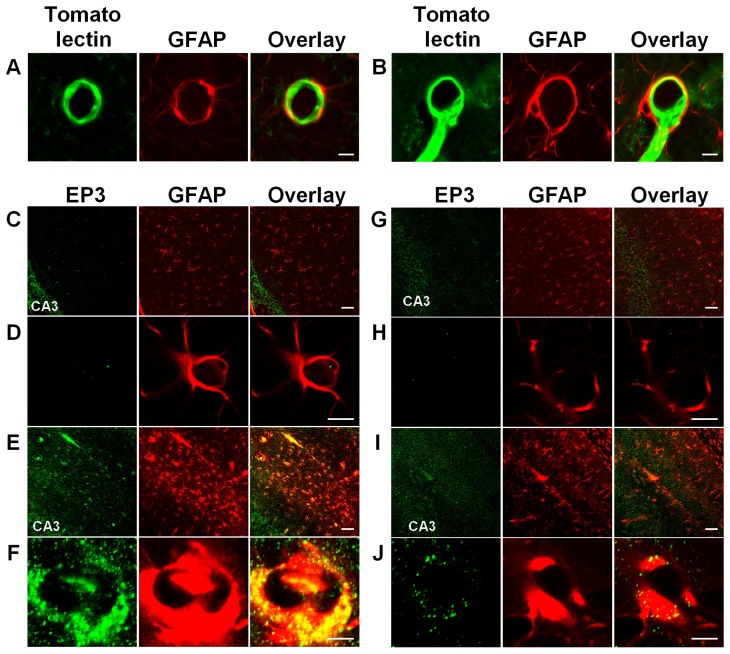
Astrocytic prostaglandin E receptor (EP)-3 is increased after KA microinjection (reprinted from ref. [19]). (**A** and **B**) Double staining with tomato lectin and an anti-GFAP antibody in the hippocampi of naive WT (**A**) and *mPGES-1*^−/−^ mice (**B**). (**C**–**J**) Double staining with anti-EP3 and anti-GFAP antibodies in the hippocampal CA3 region of naive WT mice (**C**,**D**), WT mice at 24 h after KA microinjection (**E**,**F**), naive *mPGES-1*^−/−^ mice (**G**,**H**) and *mPGES-1*^−/−^ mice at 24 h after KA microinjection (**I**, **J**). Scale bars: 5 μm in **A**,**B**,**D**,**F**,**H**,**J**; 50 μm in **C**,**E**,**G**,**I**.

Astrocytic end-feet, shown by the GFAP-stained ring-like structures surround the blood vessels (Figure 5A,B), indicating that endothelial PGE_2_ directly binds to the PGE_2_ receptor on astrocytic end-feet. We identified the PGE_2_ receptor subtype responsible for the endothelial regulation of astrocytes. We examined the effects of each EP receptor antagonist and agonist on the number of Fluo-4-labeled astrocytes in the KA-treated WT and *mPGES-1*^−/−^ slice cultures. ONO-AE3-240 (an EP3 receptor antagonist) [33] decreased the number of Fluo-4 labeled astrocytes in the KA-treated WT slices [32]. Conversely, ONO-AE-248 (an EP3 receptor agonist) [33] increased the number of Fluo-4 labeled astrocytes in the KA-treated *mPGES-1*^−/−^ slices, suggesting that the EP3 receptor has a crucial role in astrocytic Ca^2+^ elevation [32].

EP3 immunoreactivity is rarely detected in the end-feet in naive mice (Figure 5C,D,G,H); however, it is enhanced in the WT end-feet with swelling after KA injection (Figure 5E,F). In the *mPGES-1*^−/−^ mice, the end-feet also show swelling, but the EP3 immunoreactivity is not increased as much as in the WT mice (Figure 5I,J). These results indicate that the EP3 receptor is locally induced by KA in hippocampal astrocytes, which may receive PGE_2_ from endothelial cells. Preceding publications have already shown that *EP3* mRNA is expressed in cultured astrocytes [34], and EP3 protein is induced in astrocytomas by interleukin-1β [35]. These findings indicate that astrocytic EP3 receptors may be upregulated under pathological conditions, and endothelial PGE_2_ may directly activate EP3 receptors on astrocytic end-feet, not distant neuronal EP receptors in neurotoxic brain diseases, such as epileptic seizures.

### 3.4. Enhanced Glutamate Release and Neuronal Damage by Endothelial PGE_2_

In addition to astrocytic [Ca^2+^]_i_ elevation, we observed that the level of glutamate release was drastically enhanced in the WT slices by KA for 17 h, but not in the *mPGES-1*^−/−^ slices [32]. To verify whether mPGES-1 regulates hippocampal neuronal death via glutamate release, we stained the cells with propidium iodide (PI) and then calculated the fluorescence ratio of the PI uptake (*F_p_/F_pi_*) by dividing the fluorescence within the region of interest by that of the unstained region. The results show a greater degree of PI incorporation into the CA3 region of the WT slices than into that of the *mPGES-1*^−/−^ slices [32]. This significant increase in *F_p_/F_pi_* in the WT slices suggests that neuronal injury may be enhanced by mPGES-1, which controls the Ca^2+^-dependent glutamate release from astrocytes.

To validate the above findings on the endogenous PGE_2_, we added exogenous PGE_2_ to the *mPGES-1*^−/−^ slices. The application of 5 μM PGE_2_ enhanced the astrocytic [Ca^2+^]_i_ levels, particularly around the CA3 stratum radiatum, and also increased the number of Fluo-4 labeled astrocytes [32]. Moreover, PGE_2_ caused an increase in the glutamate concentration and exacerbated the neuronal damage in the CA3 region, but not in the CA1 region [32]. These results indicate that the PGE_2_ derived from mPGES-1 modulates KA-induced neuronal injury by elevating the astrocytic [Ca^2+^]_i_ levels. Moreover, we confirmed whether exogenous PGE_2_ increases the [Ca^2+^]_i_ levels in cultured neurons, because a [Ca^2+^]_i_ increase in neurons might cause neuronal damage. We found that PGE_2_ could raise the [Ca^2+^]_i_ levels in neurons co-cultured with astrocytes, but not without astrocytes (unpublished data). In addition, the [Ca^2+^]_i_ increase in neurons was found to follow the [Ca^2+^]_i_ increase in astrocytes (unpublished data). These results suggest that PGE_2_ indirectly increases the neuronal [Ca^2+^]_i_ levels via the astrocytic [Ca^2+^]_i_ increase and subsequent glutamate release. Finally, we tested whether this PGE_2_-evoked glutamate release from astrocytes occurs in a Ca^2+^-dependent manner.

### 3.5. Ca^2+^-Dependent Glutamate Release

A membrane-permeable Ca^2+^ chelator, BAPTA-AM, was applied to the slices. BAPTA-AM diminished the increase in the [Ca^2+^]_i_ levels in the astrocytes in the WT slice and abolished the increase in glutamate concentration [32]. Moreover, BAPTA-AM partially ameliorated the neuronal damage in the CA3 region, but not in the CA1 region, suggesting that CA3 neuronal damage is locally regulated by Ca^2+^-dependent glutamate release from neighboring astrocytes [32].

**Figure 6:**
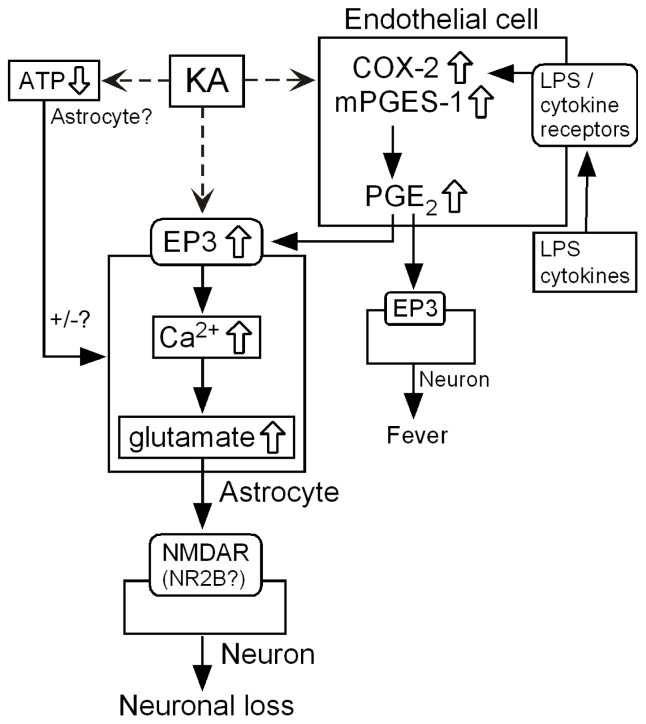
Schematic diagram of the endothelial regulation of neural damage after treatment with KA.

Taken together, these results suggest that the PGE_2_ produced by endothelial mPGES-1 activates the astrocytic EP3 receptor to elevate the [Ca^2+^]_i_ levels in astrocytes, causing Ca^2+^-dependent glutamate release and leading to neuronal injury (Figure 6) [32].

### 3.6. Intercellular Signaling among Endothelia, Astrocytes and Neurons

Accumulating evidence suggests that neuron-to-astrocyte signaling regulates arterial blood flow in the brain [22,36,37]. Conversely, there is also mounting evidence for dynamic astrocyte-to-neuron interactions, for example, astrocytes modulate synaptic transmission [28–30]. These interactions are also involved in neuronal synchrony [38] and epileptic discharges [39,40], which contribute to a delayed neuronal loss after seizures [31]. In this review, we propose an advanced mechanism for excitotoxicity via vascular endothelial cell that controls astrocyte-to-neuron signaling.

We demonstrate that endothelial mPGES-1 regulates Ca^2+^ signaling in astrocytes and Ca^2+^-dependent glutamate release from astrocytes, consistent with the finding that application of exogenous PGE_2_ propagates astrocytic Ca^2+^ waves and evokes Ca^2+^-dependent glutamate release from astrocytes [27]. However, as the application of PGE_2_ alone did not increase astrocytic [Ca^2+^]_i_ levels in our experimental system (data not shown), PGE_2_ may require another factor, such as a concomitant activation of astrocytic EP3, to elevate [Ca^2+^]_i_ levels in astrocytes after KA treatment. Furthermore, it has been shown that PGE_2_ barely increases [Ca^2+^]_i_ levels in neurons (unpublished data) and cannot evoke neuronal current directly [41], suggesting that endothelial PGE_2_ may specifically regulate astrocytes.

In addition to PGE_2_, ATP and related purine derivatives are known transmitters that are released from astrocytes, and they have been shown to depress astrocytes and neurons [42]. There is still controversy over the ATP function, because other groups claim that ATP stimulates Ca^2+^-dependent glutamate release from astrocytes via the P2Y1 receptor and propagates Ca^2+^ oscillations to neighboring astrocytes [43,44], leading to neuronal excitation. We find a reduction in the ATP concentration in the slices treated with KA for 17 h. Previous studies also showed that the systemic administration of KA causes an ATP decrease in the rat hippocampus [45,46]. Further detailed investigation is needed to infer the role of ATP in the KA-induced neurotoxicity.

Hippocampal neurons are vulnerable to glutamate, and it is thought to be mediated by *N*-methyl-d-aspartate (NMDA) receptors (NMDARs) [47]. In particular, glutamate release from astrocytes activates extrasynaptic NMDAR NR2B, which induces neuronal currents [31] or triggers neuronal cell death [31,48][49], suggesting that extrasynaptic NR2B receptors have important roles in the neurotoxicity caused by the glutamate released from astrocytes. Conversely, neuronal glutamate activates astrocytic mGluR5 to cause an increase in [Ca^2+^]_i_ levels in astrocytes, which may in turn release glutamate and feedback to extrasynaptic NMDAR NR2B [31]. Thus, the neuron-astrocyte circuit may amplify the glutamate signaling, which aggravates neuronal excitotoxicity following seizures.

The results described here indicate that brain endothelial cells are not merely a physiological barrier between the blood and brain, but may also act as a signal transducer or amplifier. In particular, endothelial cells may be active under pathological conditions, such as in epileptic seizure. In response to such insults, endothelial cells would continuously supply a large amount of PGE_2_ to astrocytes, which would in turn affect neurons. The interaction among neurons, astrocytes and endothelial cells may be a key for investigating the processes of neuropathological disorders.

## 4. Conclusions

We find that PGE_2_ is synthesized by inducible mPGES-1 in cooperation with COX-2 in vascular endothelial cells in response to KA treatment. Moreover, endothelial PGE_2_ activates astrocytic the EP3 receptor to elevate [Ca^2+^]_i_ levels in astrocytes, causing Ca^2+^-dependent glutamate release and, subsequently, stimulating neuronal damage. This is a first clarification of a mechanism for neuronal damage regulated by endothelial cells; therefore, this review emphasizes that brain endothelial cells act as a signal transducer or amplifier, especially, under pathological conditions, such as epileptic seizure. The analysis of the interactions among neurons, astrocytes and endothelial cells provides a better understanding of the processes of neuropathological disorders and will facilitate the development of new treatments.
